# Cardiac ultrasound in resource-limited settings (CURLS): towards a wider use of basic echo applications in Africa

**DOI:** 10.1186/s13089-019-0149-0

**Published:** 2019-12-27

**Authors:** Michaëla A. M. Huson, Dan Kaminstein, Daniel Kahn, Sabine Belard, Prakash Ganesh, Vanessa Kandoole-Kabwere, Claudia Wallrauch, Sam Phiri, Benno Kreuels, Tom Heller

**Affiliations:** 1 Department of Microbiology and Infectious Diseases, Erasmus Medical Centre, Rotterdam, The Netherlands; 2 Department of Emergency Medicine, Medical College of Georgia, Augusta University, Augusta, GA USA; 3 Department of Internal Medicine, University of California, Los Angeles, CA USA; 4 Department of Paediatric Pulmonology, Immunology and Intensive Care Medicine, Charité–Universitätsmedizin Berlin, Berlin, Germany; 5 Berlin Institute of Health, Berlin, Germany; 6 Lighthouse Clinic, Kamuzu Central Hospital, Lilongwe, Malawi; 7 International Training and Education Centre for Health, University of Washington, Seattle, WA USA; 8 Department of Internal Medicine, College of Medicine, University of Malawi, Blantyre, Malawi; 9 Division of Infectious Diseases and Tropical Medicine, University Hospital, LMU, Munich, Germany; 10 Department of Global Health, University of Washington, Seattle, USA; 11 Department of Medicine, University of North Carolina School of Medicine, Chapel Hill, NC USA; 12 Department of Public Health, College of Medicine, School of Public Health and Family Medicine, University of Malawi, Lilongwe, Malawi; 13 Department of Tropical Medicine, Bernhard Nocht Institute for Tropical Medicine, I. Department of Medicine, University Medical Center Hamburg-Eppendorf, Hamburg, Germany

**Keywords:** Cardiac ultrasound, Protocol, POCUS, Resource-limited setting, Africa

## Abstract

**Background:**

Point-of-care ultrasound is increasingly being used as a diagnostic tool in resource-limited settings. The majority of existing ultrasound protocols have been developed and implemented in high-resource settings. In sub-Saharan Africa (SSA), patients with heart failure of various etiologies commonly present late in the disease process, with a similar syndrome of dyspnea, edema and cardiomegaly on chest X-ray. The causes of heart failure in SSA differ from those in high-resource settings. Point-of-care ultrasound has the potential to identify the underlying etiology of heart failure, and lead to targeted therapy. Based on a literature review and weighted score of disease prevalence, diagnostic impact and difficulty in performing the ultrasound, we propose a context-specific cardiac ultrasound protocol to help differentiate patients presenting with heart failure in SSA.

**Results:**

Pericardial effusion, dilated cardiomyopathy, cor pulmonale, mitral valve disease, and left ventricular hypertrophy were identified as target conditions for a focused ultrasound protocol in patients with cardiac failure and cardiomegaly in SSA. By utilizing a simplified 5-question approach with all images obtained from the subxiphoid view, the protocol is suitable for use by health care professionals with limited ultrasound experience.

**Conclusions:**

The “Cardiac ultrasound for resource-limited settings (CURLS)” protocol is a context-specific algorithm designed to aid the clinician in diagnosing the five most clinically relevant etiologies of heart failure and cardiomegaly in SSA. The protocol has the potential to influence treatment decisions in patients who present with clinical signs of heart failure in resource-limited settings outside of the traditional referral institutions.

## Background

Point-of-care ultrasound is increasingly being used as a diagnostic tool in resource-limited settings. With more portable and affordable machines, providers from small clinics to large hospitals have increasing access to this technology. The majority of ultrasound protocols aimed at cardiac pathology have been developed and implemented in high-resource settings where more advanced imaging technology is usually available to help the clinician confirm their ultrasound diagnosis. As cardiovascular disease is an increasing cause of morbidity and mortality in sub-Saharan Africa (SSA) [1], there is a need for context-specific ultrasound protocols suited to the needs and available resources in this setting.

In SSA, patients with heart disease often present late in their clinical course with signs and symptoms of heart failure [2]. The etiology of heart disease in this context falls into several categories which are difficult to distinguish at the end stages, including hypertension and coronary artery disease [3], as well as communicable diseases such as tuberculosis (TB) and rheumatic fever [3]. There are striking differences in the epidemiology of heart diseases when comparing SSA with the United States or Europe [4]. Based on a number of studies comparing patients from SSA with patients from Europe, the Americas and Asia, African patients with heart failure are younger, more likely to be female and less likely to suffer from ischemic heart disease [4–6].

Patients with heart failure of various etiologies commonly present with a similar syndrome of dyspnea, edema and cardiomegaly on chest X-ray. In tropical settings common cardiac causes of dyspnea and edema are dilated cardiomyopathy (e.g., peripartum or HIV-associated cardiomyopathy), hypertensive cardiomyopathy, pericardial effusion with tamponade (most commonly due to TB), post-rheumatic heart disease (RHD) and cor pulmonale [7, 8]. Point-of-care ultrasound may be the only tool available to clinicians to help them make definitive diagnoses allowing for targeted treatment.

A large body of evidence has demonstrated the feasibility and clinical utility of point-of-care cardiac ultrasound performed by non-cardiologists [9–13]. In SSA, studies on handheld echocardiography showed promising results in screening children for RHD by both cardiologists and non-experts, trained in a simplified screening protocol [9–11, 14, 15]. In addition, focused cardiopulmonary ultrasound was demonstrated to improve diagnostic accuracy in emergency care in Ghana, particularly in patients with cardiac disease such as cardiogenic shock, congestive heart failure or acute valvular disease [16]. More widespread use of echocardiography in low- and middle-income countries beyond referral centers has great potential. A recent study performed in a district hospital in South Africa demonstrated that patient management was influenced by echocardiography in 83.8% of patients, who predominantly suffered from valvular heart disease [17]. A study in Rwanda demonstrated that trained nurses were able to accurately diagnose different causes of heart failure and provide care for heart failure patients using a simplified echocardiography protocol [18]. Similarly clinical officers in Kenya were able to improve their diagnosis of cardiac conditions using a focused protocol after an 8-h training [12]. These studies demonstrate the numerous applications and practical possibilities to expand the use of echocardiography in SSA.

As ultrasound becomes more ubiquitous, there is a need for context-specific training protocols. In the following pages, we outline a cardiac ultrasound protocol specifically for providers working in sub-Saharan Africa (SSA). This protocol is based on a literature review of locally prevalent conditions, and created for ease of use both in regards to performing ultrasound with basic B mode software capabilities and for teaching cardiac ultrasound in a way that is most relevant for healthcare professionals in SSA.

## Methods

### Literature review on prevalence of cardiac conditions

We searched PubMed with the following search string: “Africa” [MeSH Terms] AND “heart diseases” [MeSH Terms] and extracted information on the frequency of causes of heart failure. Our search was conducted on 1 August 2018, and an additional search on 11 October 2019. We limited our search to papers published in the last 5 years and relevant references cited herein published within the last 10 years. We excluded papers focusing on pediatric populations, ECG findings, surgery, cardiovascular risk or stroke, studies outside SSA, case reports and studies focusing on clinical characteristics of specific groups, such as HIV patients or patients with congenital heart disease. Review papers were included to search for additional relevant references. Geographic area, number of assessed patients and relative frequencies of underlying conditions were extracted. These data were subsequently used to identify the most relevant conditions that need to be assessed in a cardiac ultrasound protocol.

### Weighting of cardiac ultrasound application

Given that our goal is to create a protocol that is most relevant for clinicians in resource-limited settings, epidemiology was combined with ease of use and predicted clinical relevance to create a scoring system that would identify the high-yield ultrasound applications. Each application was assessed on three components: disease prevalence, diagnostic impact, and ultrasound difficulty (Table 1). The weight of disease prevalence was based on data from our literature review. We considered approximate average frequencies above 15% as frequent (weight = 3) and below 5% as rare (weight = 1). As the populations under study varied in age and sex distribution, and may thus not be comparable, no overall average prevalence was calculated. Prevalence was weighted as 3 if the prevalence was > 15% in the majority of studies. Weighting ultrasound difficulty was based on expert consensus between co-authors with experience in ultrasound teaching in resource-limited settings (TH, DKam, MH, VK, BK); weight of therapeutic impact by consensus of co-authors experienced in treating cardiac patients in clinical practice in these settings (DKah, PG, CW). Although the multifactorial weighting model has not been formally validated and the weights are partially subjective, the method first proposed by Van Hoving et al. [19] is transparent and easy to apply. Therefore, we consider it currently the best option to inform decisions about relevant curriculum content in various settings. The weighting of cardiac ultrasound applications allowed us to generate a protocol for focused cardiac ultrasound in resource-limited settings (CURLS) that is based on the most relevant applications and prioritizes practicality and ease of use for non-expert sonographers.

**Table 1:** Weighting of prevalence, diagnostic impact and difficulty of POCUS applications

Weight	Disease prevalence	Diagnostic impact of US	US difficulty and technical requirements
1	Rare (< 5%)	Minor or no management change	Technically advanced, often requiring special equipment like TEE probe, cw-Doppler, cardiac software
2	Relatively common (5–15%)	Management change	Moderate, may require color-Doppler
3	Very common (> 15%)	Urgent management change (possibly life threatening)	Technically easy, only basic b/w US

For prevalence and impact the numbers indicate the following levels: 1 = low, 2 = medium, 3 = high. For difficulty, scoring is reversed with numbers indicating the following levels: 1 = high, 2 = medium, and 3 = low. This allows for a composite score where the higher numbers correspond to increasing relevance and applicability of POCUS

*US* ultrasound, *TEE* transesophageal echocardiography, *cw* continuous wave, *b/w* black and white

## Results

### Epidemiology of heart disease in sub-Saharan Africa

**Fig. 1:**
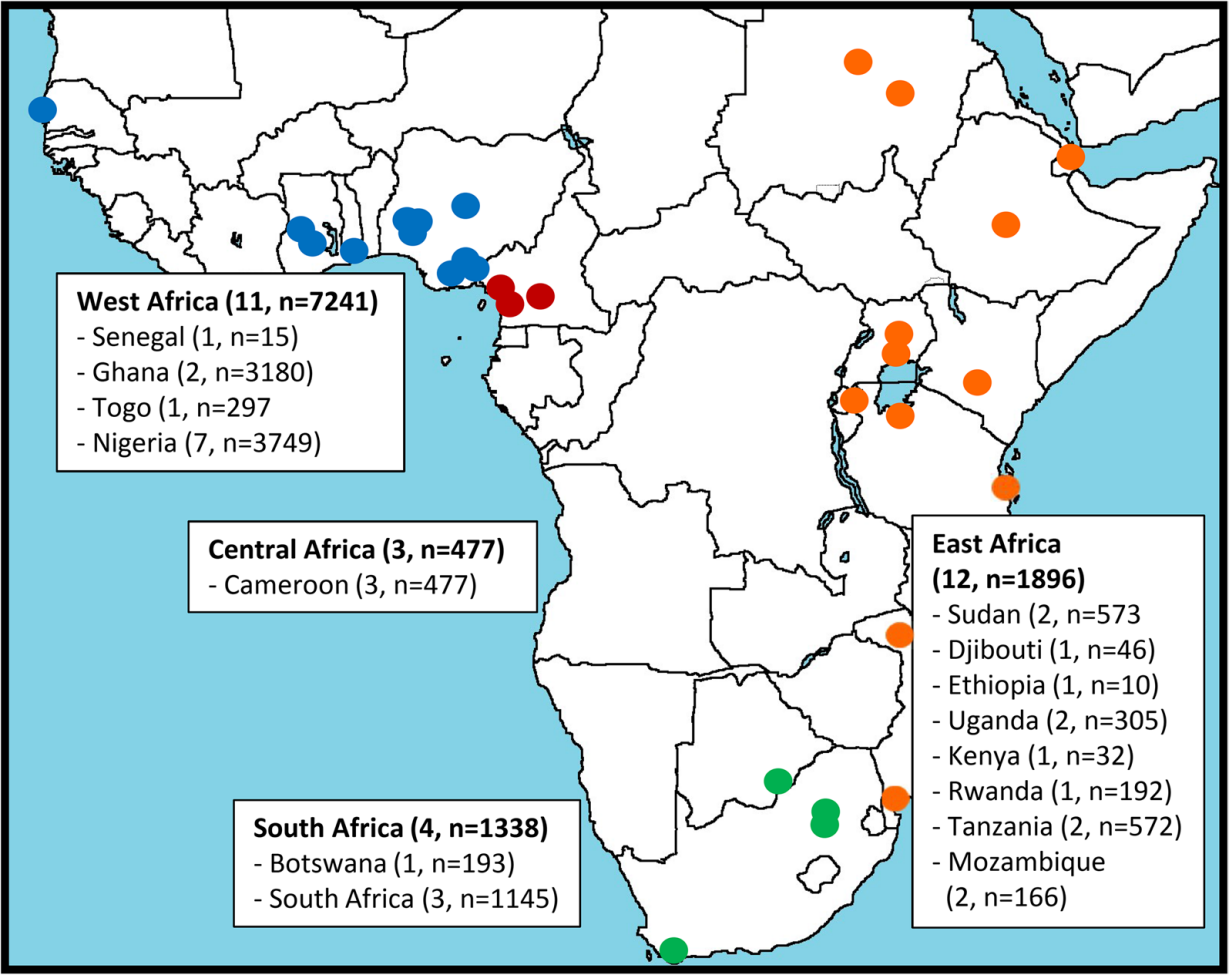
Geographic area of the 18 included studies on frequency of causes of cardiac failure. Note that some studies included multiple sites. Therefore, there are more study sites in the figure than included studies

878 titles and abstracts were screened; nine papers provided relevant information on etiologies of cardiac failure in SSA [20–28], nine additional papers were identified by reviewing references [18, 29–36]. Geographic distribution of the study areas of included studies is presented in Fig. 1, showing the wide geographic range. The literature review flowchart and baseline characteristics of included studies are given in Additional file 1: Figure S1 and Table S1. Relative frequencies of the etiologies of heart failure are summarized in Table 2. Hypertension was the most common cause of heart failure in all studies, except in one study from Djibouti where ischemic heart disease was the most common cause [32]. Cardiomyopathy was the second most common cause in most studies. Ischemic and valvular causes were less common, but reported by most studies. Endomyocardial fibrosis, pericardial effusion, cor pulmonale and congenital causes were only reported by a selected number of publications and their frequency was low.

**Table 2:** Etiology of heart failure in sub-Saharan Africa

First author, year	Patients with HF (*n*)	Hypertension (%)	Dilated cardiomyopathy (%)^a^	Ischemic (%)	Valvular (%)	Right-sided HF/cor pulmonale (%)	Effusion (%)	Endomyocardial fibrosis (%)	Congenital (%)
Ansa, 2016	339	48.6	35.4		1.4				
Appiah, 2017	1916	52.3	19.8	4	7.6				0.4
Bonsu, 2017	1488	61.2	19.9	12.9					
Boombhi, 2017	148	30.2	28.6	6.4	11.9	8.7	4		
Damasceno, 2012	1006	45.4	18.8	7.7	14.3		6.8	1.3	
Dokainish, 2017	1294	35	14.2	20	11				0.1
Kingery, 2017	588	42.8	19.3	6.2	16.6	7.6			
Kwan, 2013	192	8	54		25		1.4	0.7	5
Makubi, 2014	427	45	22.4	9	12				
Massoure, 2013	45	13	7	62					
Mwita, 2017	193	40.4	19.6	5.7	9.3		6.2		
Nkoke, 2017	529	43.2	17.6	9.6	11.7	8.8	3.8		2.1
Ogah, 2013	452	78.5	7.5	0.4	2.4	4.4	3.3	0.9	0.4
Ojji, 2013	1515	60.6	12	0.4	9.4				
Onwuchekwa, 2009	423	56.3	7.3	0.2	4.3	2.1			
Pio, 2014	297	43.1	5.9	19.2	11.8	2.7	1.7		2.7
Stewart, 2008	844	33	28	9	8	27			

*HF* heart failure

^a^The majority of this group consists of idiopathic dilated cardiomyopathy. Peripartum cardiomyopathy and HIV-related cardiomyopathy were mentioned in several studies, while thyrotoxicosis, alcohol and diabetes were mentioned incidentally as causes for dilated cardiomyopathy

### The cardiac ultrasound in resource-limited settings (CURLS) protocol

Based on the 5 highest ranking conditions (Table 3), we propose a protocol that focuses on the identification of pericardial effusion, dilated cardiomyopathy, cor pulmonale, rheumatic mitral disease, and left ventricular hypertrophy. The proposed protocol answers 5 key questions summarized in Box 1. All 5 questions can be answered using a single subxiphoid view in B mode. More advanced sonographers have the option of additional views as outlined in Table 4 (Additional files 2, 3, 4, 5, 6, 7).

**Table 3:** Ranking of cardiac ultrasound applications according to prevalence, impact and difficulty

Echocardiographic finding	Prevalence (P)	Impact (I)	Difficulty (D)	PxIxD	Rank
LV hypertrophy	3	2	2	12	1
Rheumatic mitral disease (stenosis suggested by large LA)	2	2	3	12	1
Cardiomyopathy, severe	3	2	2	12	1
Cor pulmonale	2	2	3	12	1
Pericardial effusion	1	3	3	9	2
Regurgitation (MV, AV, TV by color Doppler)	2	2	2	8	3
Rheumatic mitral stenosis (valve morphology only)	2	2	1	4	4
Rheumatic aortic stenosis (valve morphology only)	2	2	1	2	4
Endocarditis (large vegetations seen on TTE)	1	2	2	4	4
RV function grading (e.g., TAPSE)	2	2	1	4	4
Mitral stenosis grading (PHT)	2	2	1	4	4
Aortic stenosis grading (continuation equation)	2	2	1	4	4
Regional hypokinesia	2	2	1	4	4
LV function grading (e.g., ejection fraction)	3	1	1	3	5
Endocarditis (TEE)	1	2	1	2	6
Endomyocardial fibrosis	1	1	2	2	6
Congenital heart diseases	1	2	1	2	6
Pulmonary artery pressure (dTR)	2	1	1	2	6

For prevalence and impact the numbers indicate the following levels: 1 = low, 2 = medium, 3 = high. For difficulty, scoring is reversed with numbers indicating the following levels: 1 = high, 2 = medium, and 3 = low. For prevalence and impact the numbers indicate the following levels: 1 = low, 2 = medium, 3 = high. For difficulty scoring is reversed with numbers indicating the following levels: 1 = high, 2 = medium, and 3 = low. This allows for a composite score where the higher numbers correspond to increasing relevance and applicability of POCUS

*LV* left ventricular, *LA* left atrium, *MV* mitral valve, *AV* aortic valve, *TV* tricuspid valve, *TEE* transesophageal echocardiogram, *RV* right ventricle, *TAPSE* tricuspid annular plane systolic excursion, *PHT* pressure half-time, *dTR* pressure gradient measured in tricuspid regurgitation

#### Box 1: Cardiac ultrasound in resource-limited settings (CURLS) protocol—5 questions

**Table:** 

CURLS protocol: 5 questions	Interpretation
1. Is a pericardial effusion present?	Yes: consider cardiac tamponade No: consider alternative cause of heart failure
2. Is the left ventricular function reduced?	Yes: consider cardiomyopathy of various causes No: consider alternative cause of heart failure
3. Is the right ventricle larger than the left ventricle?	Yes: consider pulmonary artery hypertension or pulmonary embolism No: consider alternative cause of heart failure
4. Is the left atrium larger than the left ventricle?	Yes: consider mitral stenosis or regurgitation, possibly caused by rheumatic heart disease No: Consider alternative cause of heart failure
5. Is the left ventricle wall (septum) thicker than 12 mm?	Yes: consider hypertension, or aortic stenosis/regurgitation No: consider alternative cause of heart failure

**Table 4:** Clinical signs of heart failure or cardiomegaly on chest X-ray: narrowing differential diagnoses by cardiac ultrasound to guide management/and respective management (video clips of a normal heart and for each pattern are available as Additional file 2)

Ultrasound image	Effusion	Dilated CMP	Right-sided heart failure	Valvular (mitral)	LV Hypertrophy
				
				
Scanning method	Subxiphoid view Optional: 4-chamber view	Subxiphoid view Optional: 4-chamber view	Subxiphoid view Optional: 4-chamber view, parasternal short	Subxiphoid view Optional: 4-chamber view, parasternal long	Subxiphoid view Optional: parasternal long
Key US features	Anechoic fluid surrounding the heart^a^ In severe cases collapse of RV (tamponade)	Reduced inward movement of the LV wall Generalized dilatation of both atria and ventricles	Dilated RV in comparison to the left (ratio > 0.7) D-shaped LV in the parasternal short axis	Dilated LA Thickened mitral valve Mitral regurgitation on Doppler	Thickened LV (septum > 12 mm) Dilated LA Possibly secondary dilated right heart
Differential diagnosis	TB Malignancy Uremia Massive fluid overload Viral Auto-immune	HIV CMP Idiopathic dilated CMP (post-infectious) Peripartum CMP Alcoholic CMP Ischemic heart disease (severe)	Pulmonary embolism Pulmonary hypertension of other cause	Rheumatic heart disease	Hypertension Aortic stenosis Genetic hypertrophic CMP

*RV* right ventricle, *LV* left ventricle, *LA* left atrium, *CMP* cardiomyopathy, *TB* tuberculosis, *HIV* human immunodeficiency virus

^a^Use the parasternal long axis to differentiate between pleural and pericardial effusions. Pericardial effusions continue anterior to the descending aorta, whereas pleural effusions are found posterior to the descending aorta

#### 1. Is a pericardial effusion present?

Pericardial effusion is easily identified in a subxiphoid view and usually presents as an anechoic rim around the heart. If a significant pericardial effusion is present, the heart should be evaluated for signs of tamponade. If jugular veins are distended, the patient has tachycardia and low blood pressure, obstructive shock and tamponade may be present. Right atrial systolic collapse and right ventricle (RV) diastolic collapse suggest tamponade physiology. It is important to be aware that if the rate of fluid accumulation is rapid, even a small effusion can cause hemodynamic compromise. In a patient with cardiac tamponade emergency pericardiocentesis can be life-saving. In resource-limited settings an ultrasound-guided intercostal approach with a simple cannula can be used [37].

#### 2. Is the left ventricular function reduced?

Dilated cardiomyopathy can have multiple causes, but sonographically, the end stage is similar with global enlargement of both atria and ventricles with reduced left ventricular systolic function. Unfortunately, most symptomatic patients present in a late stage of the disease. Formal measurement of the ejection fraction and assessment of regional wall motion abnormalities is mostly confined to referral centers as it requires dedicated echocardiographic software and substantial experience. However, a broad classification of left ventricular contractility as hyperdynamic, normal, moderately impaired and severely impaired, can be made by estimating whether contraction is symmetrical towards the center, whether the myocardium thickens as it contracts, and whether the mitral valve opens normally during diastole. In an enlarged heart, this can usually be achieved in a subxiphoid view.

#### 3. Is the right ventricle larger than the left ventricle?

An enlarged RV can indicate cor pulmonale. Cardiac ultrasound can demonstrate sonographic features of cor pulmonale by evaluating shape, size and pressure of the right side of the heart. In the subxiphoid view, in the presence of a normal-sized left ventricle (LV), the RV is typically 2/3 the size of the LV, any larger suggests RV enlargement. If the RV is equal in size to the LV, it is considered moderately enlarged. Pronounced dilatation of the RV such that it is larger than the LV is considered severe RV enlargement and cor pulmonale may be considered. In addition, movement of the intraventricular septum away from the RV indicates increased right ventricular pressure or volume, resulting in a D-shaped LV in the parasternal short axis view (optional view). The measurement of RV contractility and the calculation of the pressure gradient in the setting of tricuspid regurgitation can be helpful. However, this is technically more difficult and requires Doppler software, so it is best reserved for referral sites.

#### 4. Is the left atrium larger than the left ventricle?

An enlarged left atrium (LA) is a feature of mitral stenosis and/or regurgitation, which may serve as a surrogate marker for RHD. The LA often becomes larger than the normal-sized LV by the time the patient presents. Additional findings include a thickened mitral valve with a diastolic hockey stick appearance or “doming” of the valve leaflets. The right heart may also be enlarged due to the subsequent increase in pulmonary pressures. Post-rheumatic changes of the aortic valve are slightly more difficult to detect as they initially lead to left ventricular hypertrophy only and the aortic valve is more difficult to visualize on ultrasound from a subxiphoid view. The morphological assessment of the aortic valve is possible at referral centers, as is the detection of mitral and aortic regurgitation if color-Doppler is available. For the basic CURLS exam, these questions were not included.

#### 5. Is the left ventricle wall (septum) thicker than 12 mm?

Left ventricular hypertrophy can be determined by measuring the intra-ventricular septum (IVS) at end diastole. The standard measurement of the IVS is performed in the parasternal long axis view and should be less than 12 mm. Nevertheless, the septum is generally visible in the subxiphoid view. The clinician should freeze the screen and scroll back to end diastole where the IVS is measured at the level of the mitral valve leaflet tips.

## Discussion

The clinical syndrome of heart failure is prevalent around the world. However, the underlying pathology differs significantly between resource-limited and resource-affluent settings. Ischemic heart disease is the main underlying cause in high-resource settings, but is only observed in a minority of patients in SSA. On the other hand, dilated cardiomyopathy, RHD and pericarditis (mostly TB-related) are important causes of heart failure in resource-limited settings, which are less prevalent in high-resource settings. Limited cardiac ultrasound can play a significant role in differentiating between underlying causes. Based on our analysis of the frequency of cardiac conditions, the impact on treatment decisions and the ease of use, we identified five cardiac conditions relevant to SSA for the CURLS protocol. The protocol should serve in emergency departments or acute patient settings and is not intended for screening asymptomatic patients.

Our literature search identified hypertension as the most common cause of heart failure followed by dilated cardiomyopathy and valvular causes. Pericardial effusion and cor pulmonale were less frequently reported, but play a significant role in sub-populations such as HIV-infected patients. Our suggested “cardiac ultrasound for the resource-limited setting” (CURLS) protocol aims to identify these conditions in the “5-question” approach summarized in Box 1 and Table 4. The first question addresses pericardial effusion. Although less common then the other cardiac conditions, it is very easy to recognize and can have life-threatening implications when present. In African patients effusions that mimic congestive heart failure are a common presenting sign of TB [38]. Tuberculosis is the most common cause of pericarditis in Africa. In a South African study, TB pericarditis was responsible for 70% of all cases referred for diagnostic pericardiocentesis and 96% of those who were HIV infected [39]. The second question assesses LV function as dilated cardiomyopathy was the second most common cause of heart failure in SSA in our literature review, accounting for approximately 20% of cases in most studies. Causative factors include myocarditis, HIV-associated cardiomyopathy, alcohol, nutritional deficiency, thyrotoxicosis and pregnancy. In some areas, iron overload and other metabolic factors may play a role [40]. HIV-associated cardiomyopathy is reported in 9–57% of patients who are HIV-positive in Africa [41]. The third question focuses on RV size to detect cor pulmonale. While chronic obstructive pulmonary disease and pulmonary hypertension secondary to left-sided heart failure are seen in all settings, chronic fibrocavitary TB is a frequent underlying cause in Africa [42]. In addition, HIV, schistosomiasis, and pulmonary embolism can lead to chronic pulmonary hypertension eventually resulting in cor pulmonale [43, 44]. Moreover, patients with HIV may suffer from HIV-associated pulmonary artery hypertension. The fourth question considers left atrial size to assess for sequelae of mitral stenosis or mitral regurgitation. In contrast to high-resource settings, where valvular disease is largely degenerative in origin, in Africa valvular disease is almost invariably the result of infectious disease, either directly in infective endocarditis, or indirectly, following acute rheumatic disease [45]. Whereas degenerative valvular disease afflicts mainly the elderly, RHD is encountered in young, school-age children or young females of childbearing age. The course of RHD is more rapid than in degenerative disease and predisposes to cardiac failure, secondary endocarditis, atrial fibrillation and stroke [46]. Most patients with RHD have mixed mitral valve stenosis and regurgitation [47]. Aortic regurgitation is seen in less than 50% of the patients and it is found almost always in combination with mitral valve disease; aortic stenosis is rare [48]. Finally, the protocol addresses left ventricular hypertrophy, generally the result of uncontrolled hypertension. The prevalence of hypertension is high in many African settings, and remains frequently undiagnosed and inadequately treated [49]. It is therefore a frequently identified etiology for cardiac failure in SSA [50].

Ultrasound protocols designed specifically for resource-limited settings have proven effective in SSA. Heller et al. demonstrated that the FASH (focused assessment with sonography for HIV-associated tuberculosis) exam could be efficiently taught to ultrasound-naïve clinicians over a 2-day training period [51]. The FASH exam has now become widely established as an important ultrasound exam for clinicians working in African settings with a high burden of HIV [52]. Similar success in ultrasound training has been demonstrated with the application of the 10-point yes/no checklist for various point-of-care ultrasound applications in a tertiary referral hospital in Tanzania [53]. Recently, a study in Kenya demonstrated the utility of a focused cardiac protocol applied in patient care by clinical officers [54]. Henwood et al. also demonstrated that a longitudinal ultrasound training program could detect basic cardiac abnormalities with high sensitivity and specificity when compared to experienced sonographer interpretation [55]. These studies suggest that implementation of a focused CURLS protocol is feasible.

Some limitations of the protocol should be highlighted. The protocol has not been validated in clinical practice, so its sensitivity and specificity still need to be determined. While the use of only the subxiphoid view simplifies the protocol for non-expert sonographers, it is possible that the image quality is insufficient in some patients. Furthermore, as with any focused protocol, it is not a replacement of an expert cardiology ultrasound and difficult cases should be referred for more extensive workup. Finally, no evidence is available that the protocol enhances diagnosis of cardiac pathologies in resource-limited settings. Hence, the protocol we outline here is set forward as a research agenda and discussion point to encourage all providers to think about how the incredible potential of ultrasound can become more context specific for both patients and providers. Future studies need to assess if the CURLS protocol is trainable and whether significant cardiac findings are missed in comparison to “full specialist” echo exam even though this is not widely available.

## Conclusions

Based on the epidemiology of heart disease in sub-Saharan Africa and expert consensus on difficulty and impact of cardiac sonographic examinations, we propose a protocol for screening symptomatic patients with signs of heart failure and/or cardiomegaly on chest X-ray in resource-limited settings. This protocol is unique in that uses primarily the subxiphoid view and is designed to have high clinical relevance whilst being easy to use for providers at the front line of patient care in these settings. The protocol assesses pericardial effusion, dilated cardiomyopathy, cor pulmonale, mitral valve disease, and left ventricular hypertrophy. The protocol has great potential to influence treatment of patients with heart failure in resource-limited settings. Future studies are needed to evaluate whether the protocol can easily be taught and implemented by clinicians, and if ultrasound findings have a positive influence on treatment decisions without missing important findings that would be observed by a specialist echocardiogram.

## Supplementary information

**Additional file 1: Figure S1.** Literature search flowchart. **Table S1.** Baseline characteristics of included studies on etiologies of cardiac disease in SSA (2008–2018).

**Additional file 2.** Normal heart.

**Additional file 3.** Pericardial effusion.

**Additional file 4.** Dilated cardiomyopathy.

**Additional file 5.** Right sided heart failure.

**Additional file 6.** Rheumatic heart disease with mitral valve dysfunction.

**Additional file 7.** Left ventricular hypertrophy.

## Data Availability

Data sharing is not applicable to this article as no datasets were generated or analyzed during the current study.

## Abbreviations

AV: aortic valve
CMP: cardiomyopathy
CURLS: cardiac ultrasound for resource-limited settings
cw: continuous wave
dTR: pressure gradient measured in tricuspid regurgitation
FASH: focused assessment with sonography for HIV-associated tuberculosis
HF: heart failure
HIV: human immunodeficiency virus
LA: left atrium
LV: left ventricle
MV: mitral valve
PHT: pressure half-time
POCUS: point-of-care ultrasound
RHD: rheumatic heart disease
RV: right ventricle
SSA: sub-Saharan Africa
TAPSE: tricuspid annular plane systolic excursion
TB: tuberculosis
TEE: transesophageal echocardiography
TV: tricuspid valve
US: ultrasound

## References

[1] Mensah GA, Roth GA, Sampson UK, Moran AE, Feigin VL, Forouzanfar MH (2015). Mortality from cardiovascular diseases in sub-Saharan Africa, 1990–2013: a systematic analysis of data from the Global Burden of Disease Study 2013. Cardiovasc J Afr..

[2] Sliwa K, Wilkinson D, Hansen C, Ntyintyane L, Tibazarwa K, Becker A (2008). Spectrum of heart disease and risk factors in a black urban population in South Africa (the Heart of Soweto Study): a cohort study. Lancet.

[3] Sliwa K (2016). The heart of Africa: succeeding against the odds. Lancet.

[4] Sliwa K (2013). Is all heart failure the same around the globe?. Eur Heart J.

[5] Dokainish H, Teo K, Zhu J, Roy A, AlHabib KF, ElSayed A (2017). Global mortality variations in patients with heart failure: results from the International Congestive Heart Failure (INTER-CHF) prospective cohort study. Lancet Glob Health..

[6] Makubi A, Hage C, Sartipy U, Lwakatare J, Janabi M, Kisenge P (2016). Heart failure in Tanzania and Sweden: Comparative characterization and prognosis in the Tanzania Heart Failure (TaHeF) study and the Swedish Heart Failure Registry (SwedeHF). Int J Cardiol.

[7] Heller T, Mtemang’ombe EA, Huson MA, Heuvelings CC, Bélard S, Janssen S (2017). Ultrasound for patients in a high HIV/tuberculosis prevalence setting: a needs assessment and review of focused applications for Sub-Saharan Africa. Int J Infect Dis..

[8] Mayosi BM (2007). Contemporary trends in the epidemiology and management of cardiomyopathy and pericarditis in sub-Saharan Africa. Heart.

[9] Beaton A, Lu JC, Aliku T, Dean P, Gaur L, Weinberg J (2015). The utility of handheld echocardiography for early rheumatic heart disease diagnosis: a field study. Eur Heart J Cardiovasc Imaging..

[10] Ploutz M, Lu JC, Scheel J, Webb C, Ensing GJ, Aliku T (2016). Handheld echocardiographic screening for rheumatic heart disease by non-experts. Heart.

[11] Sims Sanyahumbi A, Sable CA, Karlsten M, Hosseinipour MC, Kazembe PN, Minard CG (2017). Task shifting to clinical officer-led echocardiography screening for detecting rheumatic heart disease in Malawi, Africa. Cardiol Young.

[12] Barasa E, Rogo K, Mwaura N, Chuma J (2018). Kenya National Hospital Insurance Fund Reforms: implications and lessons for universal health coverage. Health Syst Reform.

[13] Nelson BP, Sanghvi A (2013). Point-of-care cardiac ultrasound: feasibility of performance by noncardiologists. Glob Heart.

[14] Beaton A, Aliku T, Okello E, Lubega S, McCarter R, Lwabi P (2014). The utility of handheld echocardiography for early diagnosis of rheumatic heart disease. J Am Soc Echocardiogr.

[15] Lu JC, Sable C, Ensing GJ, Webb C, Scheel J, Aliku T (2015). Simplified rheumatic heart disease screening criteria for handheld echocardiography. J Am Soc Echocardiogr.

[16] Becker TK, Tafoya CA, Osei-Ampofo M, Tafoya MJ, Kessler RA, Theyyunni N (2017). Cardiopulmonary ultrasound for critically ill adults improves diagnostic accuracy in a resource-limited setting: the AFRICA trial. Trop Med Int Health.

[17] Bedeker WF, Lachman AS, Borkum M, Hellenberg D, Cupido CS (2015). Impact of transthoracic echocardiography at district hospital level. S Afr Med J.

[18] Kwan GF, Bukhman AK, Miller AC, Ngoga G, Mucumbitsi J, Bavuma C (2013). A simplified echocardiographic strategy for heart failure diagnosis and management within an integrated noncommunicable disease clinic at district hospital level for sub-Saharan Africa. JACC Heart Fail..

[19] van Hoving DJ, Lamprecht HH, Stander M, Vallabh K, Fredericks D, Louw P (2013). Adequacy of the emergency point-of-care ultrasound core curriculum for the local burden of disease in South Africa. Emerg Med J..

[20] Appiah LT, Sarfo FS, Agyemang C, Tweneboah HO, Appiah NABA, Bedu-Addo G (2017). Current trends in admissions and outcomes of cardiac diseases in Ghana. Clin Cardiol.

[21] Bonsu KO, Owusu IK, Buabeng KO, Reidpath DD, Kadirvelu A (2017). Clinical characteristics and prognosis of patients admitted for heart failure: a 5-year retrospective study of African patients. Int J Cardiol.

[22] Damasceno A, Mayosi BM, Sani M, Ogah OS, Mondo C, Ojji D (2012). The causes, treatment, and outcome of acute heart failure in 1006 Africans from 9 countries. Arch Intern Med.

[23] Dokainish H, Teo K, Zhu J, Roy A, AlHabib KF, ElSayed A (2016). Heart failure in Africa, Asia, the Middle East and South America: The INTER-CHF study. Int J Cardiol.

[24] Makubi A, Hage C, Lwakatare J, Kisenge P, Makani J, Ryden L (2014). Contemporary aetiology, clinical characteristics and prognosis of adults with heart failure observed in a tertiary hospital in Tanzania: the prospective Tanzania Heart Failure (TaHeF) study. Heart.

[25] Mwita JC, Dewhurst MJ, Magafu MG, Goepamang M, Omech B, Majuta KL (2017). Presentation and mortality of patients hospitalised with acute heart failure in Botswana. Cardiovasc J Afr.

[26] Nkoke C, Makoge C, Dzudie A, Mfeukeu LK, Luchuo EB, Menanga A (2017). A predominance of hypertensive heart disease among patients with cardiac disease in Buea, a semi-urban setting, South West Region of Cameroon. BMC Res Notes.

[27] Ojji D, Stewart S, Ajayi S, Manmak M, Sliwa K (2013). A predominance of hypertensive heart failure in the Abuja Heart Study cohort of urban Nigerians: a prospective clinical registry of 1515 de novo cases. Eur J Heart Fail.

[28] Pio M, Afassinou Y, Pessinaba S, Baragou S, N’Djao J, Atta B (2014). Epidemiology and etiology of heart failure in Lome. Pan Afr Med J.

[29] Ansa V, Otu A, Oku A, Njideoffor U, Nworah C, Odigwe C (2016). Patient outcomes following after-hours and weekend admissions for cardiovascular disease in a tertiary hospital in Calabar, Nigeria. Cardiovasc J Afr.

[30] Boombhi J, Moampea M, Kuate L, Menanga A, Hamadou B, Kingue S (2017). Clinical pattern and outcome of acute heart failure at the Yaounde Central Hospital. Open Access Lib J.

[31] Kingery JR, Yango M, Wajanga B, Kalokola F, Brejt J, Kataraihya J (2017). Heart failure, post-hospital mortality and renal function in Tanzania: a prospective cohort study. Int J Cardiol.

[32] Massoure PL, Roche NC, Lamblin G, Topin F, Dehan C, Kaiser E (2013). Heart failure patterns in Djibouti: epidemiologic transition. Med Sante Trop.

[33] Ogah OS, Stewart S, Falase AO, Akinyemi JO, Adegbite GD, Alabi AA (2014). Contemporary profile of acute heart failure in Southern Nigeria: data from the Abeokuta Heart Failure Clinical Registry. JACC Heart Fail.

[34] Ogah OS, Adegbite GD, Akinyemi RO, Adesina JO, Alabi AA, Udofia OI (2008). Spectrum of heart diseases in a new cardiac service in Nigeria: an echocardiographic study of 1441 subjects in Abeokuta. BMC Res Notes.

[35] Onwuchekwa AC, Asekomeh GE (2009). Pattern of heart failure in a Nigerian teaching hospital. Vasc Health Risk Manag.

[36] Stewart S, Wilkinson D, Hansen C, Vaghela V, Mvungi R, McMurray J (2008). Predominance of heart failure in the Heart of Soweto Study cohort: emerging challenges for urban African communities. Circulation.

[37] Heller T, Lessells RJ, Wallrauch C, Brunetti E (2010). Tuberculosis pericarditis with cardiac tamponade: management in the resource-limited setting. Am J Trop Med Hyg.

[38] Desai HN (1979). Tuberculous pericarditis. A review of 100 cases. S Afr Med J.

[39] Reuter H, Burgess LJ, Doubell AF (2005). Epidemiology of pericardial effusions at a large academic hospital in South Africa. Epidemiol Infect.

[40] Sliwa K, Damasceno A, Mayosi BM (2005). Epidemiology and etiology of cardiomyopathy in Africa. Circulation.

[41] Ntsekhe M, Hakim J (2005). Impact of human immunodeficiency virus infection on cardiovascular disease in Africa. Circulation.

[42] Aderaye G (2004). Causes and clinical characteristics of chronic cor-pulmonale in Ethiopia. East Afr Med J.

[43] Klein SK, Slim EJ, de Kruif MD, Keller TT, ten Cate H, van Gorp EC (2005). Is chronic HIV infection associated with venous thrombotic disease? A systematic review. Neth J Med.

[44] Ramlakhan R, Andronikou S, Rajkumar A (2017). The prevalence and radiological findings of pulmonary embolism in HIV-positive patients referred for computed tomography pulmonary angiography in the Western Cape of South Africa. Cardiovasc J Afr.

[45] Strasser T (1980). Community control of rheumatic heart disease in developing countries: 1 a major public health problem. WHO Chron.

[46] Essop M, Nkomo V (2005). Rheumatic and nonrheumatic valvular heart disease: epidemiology, management, and prevention in Africa. Circulation.

[47] Malla R, Thapaliya S, Gurung P, Bogati A, Khadka S, Shrestha S (2016). Patterns of Valvular Involvement in Rheumatic Heart Disease patients taking Benzathine Penicillin at Shahid Gangalal National Heart Centre, Kathmandu, Nepal. Nepal Heart J.

[48] Faheem M, Hafizullah M, Gul A, Ullah Jan H, Asghar Khan M (2007). Pattern of valvular lesions in rheumatic heart disease. J Postgrad Med Inst.

[49] Addo J, Smeeth L, Leon DA (2007). Hypertension in sub-saharan Africa: a systematic review. Hypertension.

[50] Amoah AG, Kallen C (2000). Aetiology of heart failure as seen from a National Cardiac Referral Centre in Africa. Cardiology.

[51] Heller T, Wallrauch C, Lessells RJ, Goblirsch S, Brunetti E (2010). Short course for focused assessment with sonography for human immunodeficiency virus/tuberculosis: preliminary results in a rural setting in South Africa with high prevalence of human immunodeficiency virus and tuberculosis. Am J Trop Med Hyg.

[52] Salmon M, Landes M, Hunchak C, Paluku J, Malemo Kalisya L, Salmon C (2017). Getting it right the first time: defining regionally relevant training curricula and provider core competencies for point-of-care ultrasound education on the african continent. Ann Emerg Med.

[53] Stanley A, Wajanga BM, Jaka H, Purcell R, Byrne L, Williams F (2017). The impact of systematic point-of-care ultrasound on management of patients in a resource-limited setting. Am J Trop Med Hyg.

[54] Barasa FB, DeLong A, Kimaiyo S, Koech M, Binanay C, Foster M (2018). Diagnostic accuracy of focused cardiac ultrasound for common cardiac conditions in a Kenyan hospital. J Kenya Assoc Phys.

[55] Henwood PC, Mackenzie DC, Liteplo AS, Rempell JS, Murray AF, Leo MM (2017). Point-of-care ultrasound use, accuracy, and impact on clinical decision making in rwanda hospitals. J Ultrasound Med.

