# Supplementary material for: Cardiac ultrasound in resource-limited settings (CURLS): towards a wider use of basic echo applications in Africa
Source: Ultrasound J. 2019 Dec 27;11:34. doi: 10.1186/s13089-019-0149-0 (PMC6934640; doi:10.1186/s13089-019-0149-0)
Supplement: Supplementary file 1 — Additional file 1: Figure S1. Literature search flowchart. Table S1. Baseline characteristics of included studies on etiologies of cardiac disease in SSA (2008–2018). [file 13089_2019_149_MOESM1_ESM.docx]

**Additional file**

Additional Figure S1: Literature search flowchart

**
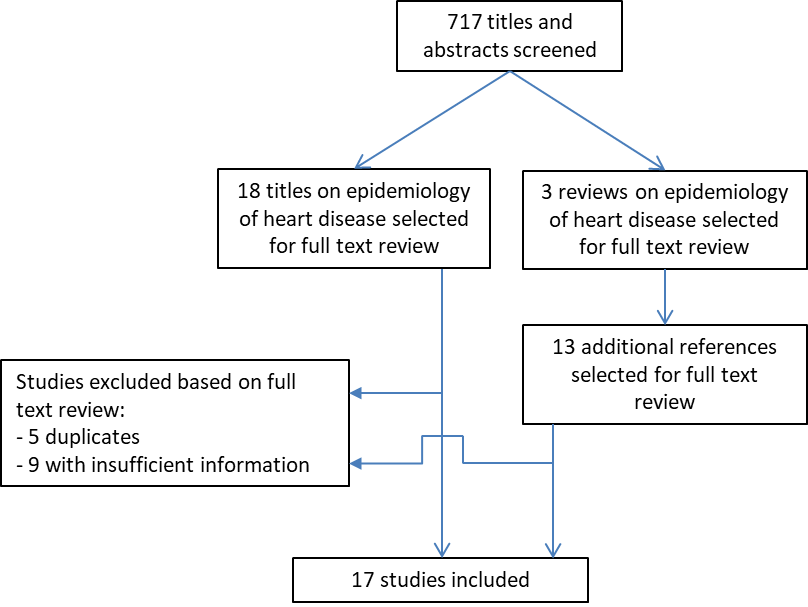
**

Additional Table S1: Baseline characteristics of included studies on etiologies of cardiac disease in SSA (2008-2018)

| **First author, year** | **Country** | **Setting** | **Design** | **Inclusion criteria** | **Male  %** | **Mean age** | **Number  (% HF)^a^** |
| --- | --- | --- | --- | --- | --- | --- | --- |
| Ansa, 2016 | Nigeria | Cardiology department of teaching hospital | RS | Patients >17 with cardiovascular admissions including stroke | 39 | 55**^b^** | 339 (43) |
| Appiah, 2017 | Ghana | Referral/teaching hospital | RS | Adult patients with cardiac admission | 52 | 59 | 1916 (88) |
| Bonsu, 2017 | Ghana | Cardiology department of teaching hospital | RS | Patients >17 with first discharge for HF | 46 | 60 | 1488 (100) |
| Boombhi, 2017 | Cameroon | Cardiology department of teaching hospital | RS | Patients >15 admitted with acute HF | 43 | 61 | 148 (100) |
| Damasceno, 2012 | Multicentre**^c^** | Referral/teaching hospital | POS | Patients >12 admitted with acute HF | 49 | 52 | 1006 (100) |
| Dokainish, 2017 | Multicentre**^d^** | Hospital and outpatient clinic | POS | Patients >17 with HF | 52 | 53 | 1294 (100) |
| Kingery, 2017 | Tanzania | Public hospital | POS | Patients >17 admitted to medical ward | 38 | 51 | 588 (25) |
| Kwan, 2013 | Rwanda | District hospital | POS | Patients (all ages) with HF | 30 | 35**^b^** | 192 (100) |
| Makubi, 2014 | Tanzania | Cardiology department of teaching hospital | POS | Patients >17 with HF | 49 | 55 | 427 (100) |
| Massoure, 2013 | Djibouti | Referral/teaching hospital | RS | Adults admitted with HF | 84 | 55 | 45 (100) |
| Mwita, 2017 | Botswana | Referral/teaching hospital | POS | Patients >17 admitted with acute HF | 54 | 54 | 193 (100) |
| Nkoke, 2017 | Cameroon | Semi urban regional hospital | RS | Patients (all ages) undergoing echocardiography | 43 | 58 | 529 (45) |
| Ogah, 2013 | Nigeria | Referral/teaching hospital | POS | Adult patients with acute HF | 55 | 56 | 452 (100) |
| Ojji, 2013 | Nigreria | Cardiology department of teaching hospital | POS | First referral to cardiology clinic | 49 | 49 | 1515 (31) |
| Onwuchekwa, 2009 | Nigeria | Referral/teaching hospital | RS | Patients >17 admitted with HF | 57 | 54 | 423 (100) |
| Pio, 2014 | Togo | Cardiology department of teaching hospital | RS | Admission with HF | 48 | 52 | 297 (100) |
| Stewart , 2008 | South Africa | Cardiology department of teaching hospital | POS | Patients with HF | 43 | 55 | 844 (100) |

**^a^** Only patients with heart failure were included in further analyses.
**^b^** In these studies a median age was provided.
**^c^** Including patients from Senegal, Nigeria, Cameroon, South Africa, Mozambique, Uganda, Kenya, Ethiopia, and Sudan.
**^d^** Including patients from Mozambique, Nigeria, South-Africa, Sudan and Uganda.
Abbreviations: RS: retrospective study, POS: prospective observational study, HF: heart failure.
